# Trained innate immunity as a mechanistic link between sepsis and atherosclerosis

**DOI:** 10.1186/s13054-014-0645-3

**Published:** 2014-11-25

**Authors:** Siroon Bekkering, Leo AB Joosten, Mihai G Netea, Niels P Riksen

**Affiliations:** Department of Internal Medicine, Division of Vascular Medicine and Experimental Internal Medicine, Radboud University Medical Center, Geert Grooteplein 10, Nijmegen, 6525 GA The Netherlands

Cardiovascular events have been associated with previous infections in many observational studies. In the previous issue of *Critical Care*, Kaynar and colleagues [1] corroborated this association by showing in atherosclerosis-prone mice that the cecal-ligation-and-puncture (CLP) model of sepsis accelerates aortic atherosclerotic plaque formation up to 5 months after CLP. In addition, the authors observed higher circulating interleukin-6 and -10 levels and a greater infiltration of macrophages in the aortic root at 5 months after CLP. Although these findings substantiate the association between infection and atherosclerosis, the underlying mechanism of the persistent inflammation remains enigmatic.

We propose the newly described process of trained immunity as the mechanistic link between previous infection and atherosclerosis. Trained immunity denotes the non-specific immunological memory that can be activated in monocytes [2,3]. Brief exposure of monocytes to microbial products (including *Candida albicans* or its cell wall component beta-glucan) induces a long-lasting pro-inflammatory macrophage phenotype via epigenetic reprogramming mediated by histone modifications [2,3]. Further analyses revealed global pan-activation of these macrophages rather than simple skewing into M1 or M2 subtypes [3].

Given the pivotal role for monocytes/macrophages in atherosclerotic plaque development, we recently hypothesized that trained innate immunity contributes to atherosclerosis [4]. Indeed, epigenetic reprogramming of monocytes induced a long-lasting pro-atherosclerotic phenotype, characterized by increased cytokine and chemokine release and increased foam cell formation [5].

Considering these findings, we propose that epigenetic reprogramming of monocytes after CLP explains the persistent inflammation and accelerated atherosclerosis. Since epigenetic reprogramming is amenable to pharmacological modulation, further elucidation of this mechanism might provide novel treatment strategies for atherosclerosis [5].

## Authors’ response

A Murat Kaynar, Alyssa D Gregory, Steven D Shapiro and Derek C Angus

We read the letter by Bekkering and colleagues with interest. We completely agree that the mechanism behind the sustained inflammation 5 months after recovery from sepsis remains enigmatic. The goal of our article [1] was to overcome the shortcomings of prior human observational studies by constructing an experiment model in which we could make causal inference. However, although we have helped establish a causal relationship, we do not know ‘how’ it actually happens. We concur that there could be innate ‘training’ through epigenetic changes leading to augmented inflammatory response by monocytes/macrophages in the absence of ongoing infection. As reported by the group, the concept of ‘learned innate immunity’ brings the innate immunity to the limelight [2]. However, the decision tree when the innate immunity does switch from a ‘tolerant’ to an energetically costly ‘learned’ state is not clear [1,6,7].

To conclude, we also think that ‘learned immunity’ could play a role in our observations. However, is ‘learned immunity’ really of benefit to the organism? If not, why do we still carry that burden?

## Abbreviation

CLP: Cecal-ligation-and-puncture
